# Interventions for pre‐school children in foster care: A systematic review of the foster carer and system level outcomes from randomised controlled trials

**DOI:** 10.1002/jcv2.70062

**Published:** 2025-11-08

**Authors:** Camilla Biggs, Natalie Kirby, Megan Garside, Gloria Cheung, Fiona Turner, Nishant Jaiswal, Helen Minnis

**Affiliations:** ^1^ School of Health and Wellbeing University of Glasgow Glasgow UK; ^2^ Adolescent Psychiatry, Leeds and York Partnership NHS Foundation Trust Leeds UK; ^3^ Leeds and York Partnership NHS Foundation Trust Leeds UK; ^4^ Hull York Medical School York and Scarborough Teaching Hospitals NHS Foundation Trust / Honorary Research Fellow Hull UK; ^5^ Health Economics and Health Technology Assessment School of Health and Wellbeing University of Glasgow Glasgow UK; ^6^ Present address: NHS Lanarkshire Child and Adolescent Mental Health Services Udston Hospital Hamilton UK

**Keywords:** caregiver stress, caregiver wellbeing, foster care, infant mental health, interventions, placement stability, pre‐school

## Abstract

**Background:**

Children in foster care are at increased risk of experiencing cognitive, emotional and behavioural difficulties. There is a window of opportunity for early intervention associated with developmental sensitivity in the early years. Foster carers and the systems supporting them play a central role in intervention and support for the young person. This systematic review explores the effectiveness of interventions for pre‐school children in foster care, in improving carer sensitivity, stress and placement stability.

**Methods:**

Embase, Medline, CINAHL, PsycInfo and Cochrane Library were search systematically for relevant articles, including randomised controlled trials (RCTs) available by 20th January 2025. Search screening, data extraction and quality appraisal were all completed by two independent researchers and reviewed collaboratively. The quality of included articles was assessed using the Cochrane Risk of Bias (RoB2) tool and GRADE assessment procedure. Narrative synthesis with meta‐analysis for some outcomes was conducted.

**Results:**

Sixteen articles, corresponding to 12 RCTs, met inclusion criteria. The quality of data according to GRADE assessment was low for all three outcome types, and RoB high for several included articles. The articles reviewed seven different intervention types. There was evidence that interventions were effective in improving caregiver sensitivity, with the strongest evidence supporting attachment and biobehavioural catch‐up and parent child interaction therapy. There was also limited evidence that interventions may improve placements stability. Overall, there was no evidence that interventions reduce caregiver stress.

**Conclusions:**

This review demonstrates the effectiveness of interventions in improving caregiver and system level outcomes—both likely to be important mediators for change in the child. Further high‐quality research is needed to identify which interventions are most effective and in what context, with improved consistency in definition and measurement of outcomes.

## Key Points

**What's known?**

- Children in foster care are at increased risk of experiencing mental health and developmental difficulties.
- Carer and system level factors are important mediators of change in interventions for children in foster care.

**What's new?**

- This review finds evidence that psychological interventions can improve foster carer sensitivity, and some limited evidence of improvements in placement stability.
- The impact that psychological interventions for pre‐school children in foster care have on caregiver stress, is inconclusive with inconsistency in study findings.

**What's relevant?**

- There is a need for further high‐quality research in this area, including a need for larger randomised controlled trials with improved consistency in measurement and definition of caregiver and system level outcomes, particularly relating to placement stability.

## INTRODUCTION

Children living in foster care have commonly experienced maltreatment and neglect and often display emotional and behavioural challenges (National Institute for Health and Care Excellence [NICE], 2021). A meta‐analysis exploring prevalence of mental health or developmental difficulties in pre‐school age foster children identified around 39% display a developmental delay; 38% clinically significant psychological difficulties; and 43% have an insecure attachment style, most commonly disorganised attachment (Vasileva & Petermann, 2018).

The early years are a period of neurodevelopmental sensitivity, particularly in relation to the attachment system, and development of neural structures involved in emotion expression and regulation (Perry et al., 2017; Zeanah & Humphreys, 2018). Effective intervention during the early years offers an opportunity for mitigating the impact of prior maltreatment and preventing long‐term adverse outcomes (Minnis, 2024).

In addition to maltreatment prior to entering foster care, many children (20%–50%) also experience placement disruption or unplanned placement termination (Konijn et al., 2019). There is a bidirectional relationship between child behavioural problems and placement disruption, whereby externalising behaviours in particular predict placement changes, but increased placement disruption also predicts increased externalising difficulties (Maguire et al., 2024). This increased risk of placement breakdown may relate to factors including higher levels of foster carer stress, foster carers' perception of their capacity to support the child and receive sufficient support from services (Tonheim & Iversen, 2019; Turner et al., 2023; Whenan et al., 2009). Foster carers' parenting capacity has also been correlated with levels of caregiver stress, highlighting the potential vicious cycle whereby children with more behavioural needs might receive poorer caregiving (Konijn et al., 2019). Research has demonstrated that placement stability, the coordination of placement changes (e.g., sudden transitions) and foster carers relational style are correlated with child mental health outcomes (Hillen & Gafson, 2015). When considering the effectiveness of interventions aiming to improve developmental and mental health outcomes for children in foster care, it is extremely important to consider the caregiver and system level outcomes.

Previous systematic reviews have considered the effectiveness of psychosocial interventions for foster children and carers, but generally consider children of all ages, and a mixture of caregiver and child‐related outcomes. Bergstrom et al., 2020, report potential benefit of psychosocial interventions across several outcomes but does not differentiate effectiveness of different programs (Bergström et al., 2020; Schoemaker, Wentholt, et al., 2020), meta‐analysis reported effectiveness of parenting interventions in improving caregiver sensitivity, behaviour, knowledge and stress (Schoemaker, Wentholt, et al., 2020). However, both these reviews include interventions for foster children at any age.

A focus on pre‐school children, who have specific needs, is required (Minnis, 2024). This paper describes one of two systematic reviews, conducted in parallel, exploring the effectiveness of interventions for pre‐school children (age 0–7 years old/under 8) in foster care. The outcomes were separated into child or caregiver/system focused outcomes and split across two reviews due to the diversity of outcome measures captured. This review examines caregiver sensitivity, caregiver stress and placement stability. Child wellbeing, development or child‐carer attachment outcomes are considered in a separate review (Kirby et al., 2025). The difficulties targeted are frequently described in relation to the child (e.g., behavioural difficulties) many interventions target the caregiver's behaviour as the mechanism for change.

## METHODS

The systematic literature review was conducted in accordance with the Preferred Reporting Items for Systematic Review and Meta‐Analysis (PRISMA) guidelines (Moher et al., 2009) and registered on the Prospective Register of Systematic Reviews (PROSERO) database, CRD 42022367449.

### Search strategy

Abroad search strategy was developed, in consultation with a librarian, including search terms relating to: children, foster care and RCTs, see supplementary materials (Supporting Information S1: Table S1). This was conducted across the following databases: Embase, Medline, CINAHL, PsycInfo and Cochrane Library, on the 23rd June 2023. Following data extraction de‐duplication was completed (Falconer, 2018). Researcher (C.B.) completed title and abstract screening for all identified studies (*N* = 6815), and researcher (N.K.) completed screening for 20% of studies, blinded to the first screening outcome. Inter‐rater reliability on this subset of records was 0.98. Discrepancies were discussed prior to full text review of remaining articles. Full texts (*N* = 113) were screened independently by two researchers (C.B. and N.K.) with an inter‐rater reliability of 0.97, For timely but comprehensive review, an additional search was performed by author C.B. only (on 25th January 2025), to ensure no new relevant articles had been published since initial screening.

### Eligibility criteria

Published and unpublished articles identified were considered according to the following inclusion criteria:

- RCT data for any intervention (with any comparator) for children within foster care.
- All child participants were 7 years old or younger and living in foster/kinship care.
- Caregiver or system level (e.g., placement) outcome measures reported.
- Available on searched databases in English by 20^th^ January 2025

### Risk of Bias and grade of evidence

The Cochrane Risk of Bias 2.0 (RoB) tool for RCTs was used to assess the level of RoB. Each paper was appraised by two independent researchers and discrepancies resolved through discussion. GRADE quality assessment was completed for each outcome, and decisions were reviewed by a second researcher.

### Data extraction

A team (C.B., G.C., M.G., N.K.), extracted and tabulated descriptive data for each study: study title, author, date of publication, country of research, overall sample demographics, child placement type, sample size, intervention type, control group (CG) type, outcome measurement frequency, outcome measures, reported confounding factors, reported outcome. Data relating to intervention effectiveness for the relevant outcome variables was then extracted for quantitative analysis including: sample size, mean and standard deviation pre‐ and post‐ intervention for each trial arm. Authors were contacted for additional information where required.

### Data analysis

A narrative synthesis approach following SWiM guidelines (Campbell et al., 2020) was used to analyse information available regarding placement stability due to heterogeneity in definition for this outcome. A pairwise random effects meta‐analysis comparing psychological interventions described below with service as usual (SAU) was used to synthesise data for caregiver sensitivity and caregiver stress, with narrative synthesis used to incorporate studies where data available did not allow for quantitative analysis. A meta‐analysis was deemed feasible where data was available from at least two studies for the same outcome. For meta‐analysis all interventions were considered together, with subgroups analysis for different intervention types. A funnel plot analysis for heterogeneity and assessment of publication bias within the meta‐analysis is not recommended given the small number of studies included. The three outcome variables included in this review (caregiver sensitivity, caregiver stress, placement stability) were the most consistently reported care system outcomes. Secondary caregiver measures including parenting attitudes, and depression were reported in a small number of articles but not included in the review, see Table 1. Where outcome measures were reported at multiple time‐points, only pre‐ (immediately before) and post‐ (end of intervention) measures were used. For multi‐arm RCTs, the intervention arms were combined. Where the mean and variance for change was not reported, it was imputed, see equations in supplementary materials (Supporting Information S1: Table S2; Higgins et al., 2023). *Hedges g* was used as a measure of effect size (ES), deemed most suitable given the small sample size in some included studies. Meta‐analysis was conducted using SPSS Statistics, data available from corresponding author at request.

**TABLE 1:** Study characteristics.

Study (author, year)	Publication type/source	Country	Design	Intervention	Control group	Intervention target (inclusion/exclusions)	Sample size (overall, intervention, control)	Study aim and outcome measures (primary, secondary)	Outcome measure included in this review
Bick & Dozier (2013)	Journal of Infant Mental Health	USA	RCT	ABC	DEF	Foster carers caring for foster children 22 months of age or younger	*N* = 96 IG = 44 CG = 52	Aim: Assess whether ABC effectively improves caregiver sensitivity Primary outcome: Caregiver sensitivity	Caregiver sensitivity: Video recorded interactions, scored with coded likert
Raby et al. (2019)	Developmental Science	USA	RCT	ABC‐T	DEF	Children in foster care between 24 and 36 months old	*N* = 88 IG = 45 CG = 43	Aim: Assess whether ABC‐T improves language development Primary: Receptive vocabulary ability in child Mediator/secondary: Caregiver sensitivity	Caregiver sensitivity: Video recorded interactions, scored with coded likert scale adapted from Observational record of the caregiving Environment.
Van Andel et al. (2016)	American Journal of Orthopsychiatry	The Netherlands	RCT	FFI	TAU	Children under 5 years old, in foster care and expected to remain within foster care for at least 6 months. Children where there was a high risk of placement breakdown without intervention, children with cognitive or other birth deficits were excluded.	*N* = 123 IG = 65 CG = 58	Aim: FFI effect on foster carers perception, behaviour and child reactions to carer Primary: Carer: Child interaction, caregiver sensitivity and caregiver stress Secondary: Child stress	Caregiver stress: NOSI‐R
Conn et al. (2018)	Children and Youth Services Review	USA	RCT	IY(tf)	TAU	Foster carers of children aged 2–7 years (inclusive)	*N* = 33 IG = 16 CG = 17	Aim: Determine effectiveness on child behaviour, caregiver stress/attitudes, caregiver satisfaction and contributing factors. Primary: Child behaviour, parenting attitude, caregiver stress Secondary: Foster carer satisfaction, specific factors contributing to parenting skill change	Caregiver stress: PSI‐SF
Fisher et al. (2005)	Child Maltreatment	USA	RCT	EIFC	TAU	3–6 year old foster children in need of a new foster placement within the catchment area, and expected to remain within care for more than 3 months	*N* = 90 IG = 47 CG = 43	Aim: Exploring impact on placement stability RCT outcome measures: Child psychosocial, neurocognitive, emotional, academic function, caregiver parenting practice, family satisfaction and service utilisation. Also, placement experiences including permeance or breakdown. Article reports placement stability outcome data only.	Placement stability: Placement permanency or failure
Miller (2007)	Theses: University of Oregon	USA	RCT (sub‐sample analysis)	EIFC	TAU	Children aged 3–6 years, all recently entering a new foster placement	*N* = 78	Aim: Exploring placement disruption patterns. Secondary data analysis of Fisher et al., 2005 RCT data, integrated with welfare service data. Primary: Placement outcome. Secondary/mediators: Birth parental substance use, child age, maltreatment experience, child emotional and behavioural problems, developmental delays placement history	Placement stability
Fisher & Stoolmiller (2008)	Developmental Psychopathology	USA	RCT	MTFC‐P	TAU	Foster pre‐schoolers aged 3–6 years who were entering new placements in a county child welfare system in the Pacific Northwest with a planned placement of at least 3 months	*N* = 117 IG = 57 CG = 60	Aim: Determine effectiveness of interventions on caregiver stress and explore interaction with child behaviour and child HPA axis activity Primary: Caregiver stress when managing child's behaviours Secondary: Child HPA Axis activity—secondary analysis of association between caregiver stress and child HPA axis	Caregiver stress: Parent daily report data
Fisher et al. (2011)	Journal of Consulting and Clinical Psychology	USA	RCT	MTFC‐P	TAU	Foster pre‐schoolers aged 3–6 years who were entering new placements with a planned placement of at least 3 months	*N* = 117 IG = 57 CG = 60	Data from Fisher & Stoolmiller, 2008 targeting caregiver and child stress. Primary: Placement stability Mediator: Child ‘problem behaviour’	Placement stability: Placement disruptions
Fisher et al. (2009)	Child Youth Services Review	USA	RCT	MTFC‐P	TAU	Foster pre‐schoolers aged 3–6 years who were entering new placements with a planned placement of at least 3 months	*N* = 52 IG = 29 CG = 23	Primary aim: characterise placement instability against maltreatment experience Secondary aim: MTCP‐P effect on placement stability. Outcome: Placement stability.	Placement stability: Placement permanency
Jonkman et al. (2017)	Journal of Child and Family Studies	Netherlands	RCT	MTFC‐P	TAU	Children between 3 and 7 years, indicated for permanent foster care placement	*N* = 34 IG = 23 IG = 11	Aim: Explore effectiveness as intervention for children with significant behaviour problems Primary: Child behavioural problems Secondary: Child attachment, child trauma symptoms, caregiver stress, child HPA‐axis functioning	Caregiver stress: PSI‐SF
Mersky et al. (2015)	Journal of the Society for Social Work Research	USA	RCT	PCIT/PCIT extended	WLC	Children aged 3–6 years, placed in a licenced, nonrelative foster home, and in the clinical range for externalising problems on the Eyberg child‐behaviour inventory (ECBI) according to foster carer ratings. Exclusions: Children with intellectual, physical, or pervasive developmental disabilities.	*N* = 102 IG (PCIT) = 39 IG (PCIT ext.) = 19 CG = 33	Aim: Explore impact on parenting stress and behaviour from intervention designed to improve child behaviour Primary: Caregiver stress, parenting behaviours/sensitivity	Caregiver stress: PSI‐SF Caregiver sensitivity DPCICS‐II
Blair (2018)	Thesis: University of Wisconsin–Milwaukee	USA	RCT	PCIT	TAU	Children aged 3–6 years, placed in a licenced, nonrelative foster home, and in the clinical range for externalising problems on the Eyberg child‐behaviour inventory (ECBI) according to foster carer ratings. Exclusion: Children with intellectual, physical, or pervasive developmental disabilities	*N* = 123 IG = 80 CG = 43	Data from Mersky et al., 2015, targetting caregiver stress and parenting behaviours/skills. This is secondary analysis integrating child welfare data. Primary outcome: Placement strability.	Placement disruption and permanency outcomes
Danko (2014)	Thesis: DePaul University	USA	RCT	CDIT/PCIT	WLC	Foster care families with foster child between 2 and 5 years old, with child placed with foster carer for at least 2 months.	*N* = 27 IG (CDIT) = 8 IG (PCIT) = 7 CG = 9	Aim: Improve attachment relationship Primary: Child attachment to caregiver Secondary: Caregiver stress, caregiver depression, child behaviour problems	Caregiver stress: PSI‐SF
N'zi et al. (2016)	Child abuse and neglect	USA	RCT	CDIT	WLC	Kinship carers caring for a child between 2 and 7 years old, expecting the child to be residing in their home for the duration of the study and with a caregiver rating one standard deviation above the norm on Eyberg child behaviour problem Scale	*N* = 14 IG = 7 CG = 7	Primary aim: feasibility of intervention Secondary aim/primary outcomes: Efficacy for measures—child behaviour, caregiver: Child relationship quality, caregiver stress and depression, caregiver discipline and caregiver sensitivity	Caregiver stress: PSI‐SF Caregiver sensitivity: DPCICS‐IV
Job et al. (2022)	Journal of Interpersonal Violence	Germany	RCT	TCTP	TAU	Children in foster (not kinship) care for less that 24 months, aged between 2 and 7 years with a primary allegation of child maltreatment or neglect as indicated by welfare files	*N* (fam.) = 81 *N* (child) = 87 IG (fam.) = 44, IG (child) = 46 CG (fam) = 37 CG (child) = 41	Aim: Improve parent child relationship via parenting competencies. Primary: Parent‐child relationship and caregiver sensitivity Secondary: Child mental health and foster carer commitment.	Caregiver sensitivity: DPCICS‐IV and mother‐child play task observation system
Schoemaker, Juffer, et al. (2020)	Child and Youth Services Review	Netherlands	RCT	VIPP‐FC	DI	Families with foster child between 1 and 6 years, with expected duration of placement at least 6 months	*N* = 60 IG = 30 CG = 30	Aim: Improve parenting behaviour and sensitivity primary: Parenting behaviour (sensitive parenting and sensitive discipline, parent attitudes (toward sensitive parenting)	Caregiver sensitivity: Video recorded interactions, scored with coded likert scale, adapted version of Ainsworth Scale of sensitivity

*Note:* Studies relating to the same RCT.

Abbreviations: ABC, attachment and biobehavioural catch‐up; ABC‐T, attachment and biobehavioural catch‐up for toddlers; CDIT, child directed interaction training; CG, control group; DEF, developmental education for families; DI, dummy intervention; DPCICS‐IV, dyadic parent–child interaction coding system 4th edition; EIFC, early intervention foster care program; FFI, foster care—foster child intervention; HPA, hypothalamic‐pituitary‐adrenal; IG, intervention group; IY(tf), trauma‐informed adapted incredible years; MTFC‐P, multidimensional treatment foster care for pre‐schoolers; NOSI‐R, Dutch version of parenting stress index; PCIT, parent child Interaction therapy; PSI‐SF, parenting stress index short form; RCT, randomised controlled trial; TAU, treatment as usual; TPTP, triple P system for foster carers; VIPP‐FC, video‐feedback Intervention to promote positive parenting and sensitive discipline in foster care; WLC, waitlist control.

## RESULTS

### Study selection

**FIGURE 1:**
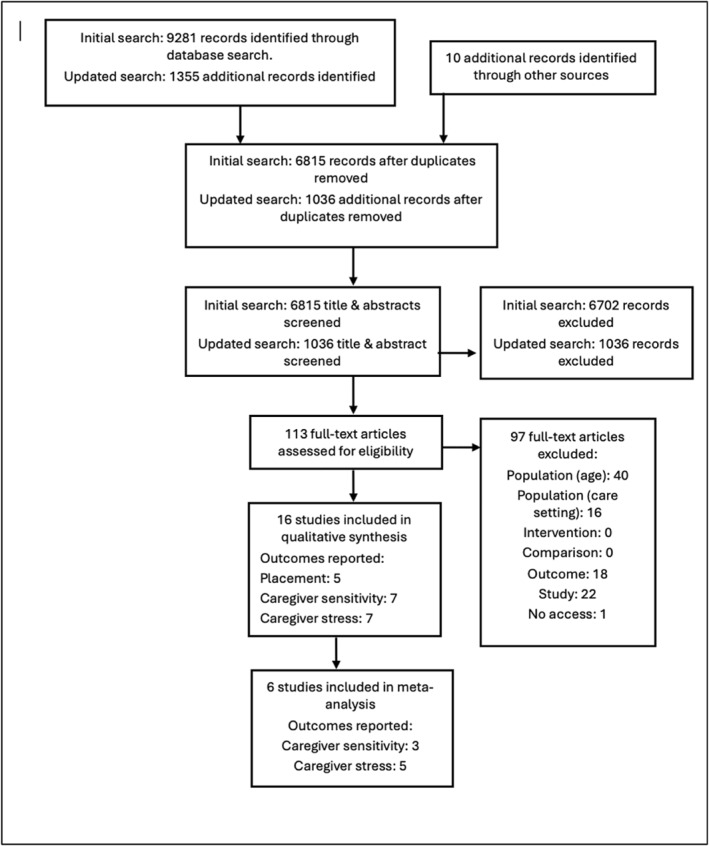
PRISMA flow diagram for search conducted. Initial search, performed on the 23.06.2023. Updated search performed on 20.01.2025.

In total, 10,636 records were identified, 7851 after de‐duplication. Seven thousand, seven hundred thirty‐eight records were excluded through title and abstract screening, and 113 excluded through full text review, leaving 16 records which met criteria for inclusion. The reasons for exclusion after full text review were: child's age, children not residing in foster care only, study design, and outcome measures not relevant. See PRISMA flow diagram (Figure 1).

### Study characteristics

The review includes 13 published research papers and 3 PhD dissertations (Blair, 2018; Danko, 2014; Miller, 2007). Twelve included records were based in the USA, 1 in Germany (Job et al., 2022) and 3 in the Netherlands ((Jonkman et al., 2017; Schoemaker, Juffer, et al., 2020; Van Andel et al., 2016). Not all included records related to different RCTs, such that the 16 included records correspond to 12 RCTs. The majority, 11 records (corresponding to 7 RCTs), compared the Intervention group (IG) with ‘services as usual’ (Bick & Dozier, 2013; Blair, 2018; Conn et al., 2018; Fisher et al., 2005, 2009, 2011; Fisher & Stoolmiller, 2008; Job et al., 2022; Jonkman et al., 2017; Mersky et al., 2015; Miller, 2007; Van Andel et al., 2016). Two records, corresponding to 2 RCTs, compared with a ‘waitlist control group’ (Danko, 2014; N’Zi et al., 2016). Three records, corresponding to 3 RCTs compared the intervention with an active ‘dummy’ intervention (Bick & Dozier, 2013; Raby et al., 2019; Schoemaker, Juffer, et al., 2020). Study characteristics are described in Table 1.

### Participants

This review includes 1034 foster children and approximately 1034 foster carers. The exact number of foster carers cannot be calculated, as stome studies refere to foster family (e.g., Danko, 2014) and one study included families with more than one foster child (Job et al., 2022), RCT sample size ranged from 14 (N’Zi et al., 2016) to 123 (Blair, 2018; Van Andel et al., 2016). The age of children ranged from 1 month (Bick & Dozier, 2013) to 95 months (Job et al., 2022; pooled mean aged 42.63 months). The gender distribution reported was relatively balanced. For demographic information reported, see supplementary materials (Supporting Information S1: Table S3).

### Interventions

*Attachment and biobehavioural catch‐up (ABC)*: Two RCTs assessed ABC considering outcome measures relating to caregiver sensitivity (Bick & Dozier, 2013; Raby et al., 2019). ABC is a 10‐session intervention intended to increase sensitivity and nurturing parenting approaches through psychoeducation and structured play with video‐based feedback. In both instances the ABC intervention was compared with Developmental Education of Families, also a 10‐session intervention including video‐based feedback but focusing on motor and cognitive development.

*Foster carer—foster child intervention (FFI)*: One RCT compared FFI with SAU, considering caregiver sensitivity and caregiver stress (Van Andel et al., 2016). FFI consists of 6 90‐min home visits in which psychoeducation and video‐based feedback is shared with the foster carer, aiming to increase caregiver sensitivity.

*Incredible years (IY)*: One RCT assessed a trauma‐informed adaptation of IY compared with SAU, considering caregiver stress (Conn et al., 2018). Carers meet for 2.5‐h sessions, weekly for 13 weeks. IY is designed to build skills in positive parenting, teaching and engaging with the child.

*Multi‐treatment foster care for pre‐schoolers (MTFC‐P)*: 3 RCTs (6 studies) assessed MTFC‐P, or the previous version of this intervention early intervention foster care (EIFC) compared with SAU. Four of these studies is placement stability (Fisher et al., 2005, 2009, 2011; Miller, 2007), and 2 considered caregiver stress (Fisher & Stoolmiller, 2008; Jonkman et al., 2017). MTFC‐P is a caregiver‐based preventative intervention delivered in 3 phases, which includes 12 h of training prior to fostering a child, and then weekly individual consultation and support for carers as well as a skills trainer meeting directly with the child. The intervention is delivered over a 9 months to 1 year period.

*Parent child interaction therapy (PCIT)*: 3 RCTs (4 studies) assessed variants of PCIT compared with SAU, considering caregiver sensitivity, caregiver stress, and placement stability (Blair, 2018; Danko, 2014; Mersky et al., 2015; N’Zi et al., 2016). PCIT consists of two stages, child‐directed interaction (CDI) and then parent‐directed interaction, in which caregiver sensitivity coaching is provided to strengthen caregiver‐child relationship and the child's behaviour/carer's approach to the child behaviour. N’Zi et al., 2016 describes an RCT for effectiveness of CDI element alone, and Danko, 2014, includes both CDI only and PCIT intervention arms. Mersky et al., 2015 also includes two intervention arms, a PCIT arm and an extended version of PCIT in which two additional contacts are provided.

*Triple P (TCTP)*: One RCT assessed TCTP for foster care compared with SAU, considering caregiver sensitivity as an outcome measure (Job et al., 2022). TCTP is a parenting group intervention promoting skills in positive parenting and management of the child's behaviour, delivered across five 2.5‐h group sessions.

*Video‐feedback intervention to promote positive parenting and sensitive discipline in foster care (VIPP‐FC)*: One RCT assessed VIPP‐FC compared with a ‘dummy intervention’, considering caregiver sensitivity as an outcome variable (Schoemaker, Juffer, et al., 2020). VIPP‐FC is delivered through 6 home visits over a 3–4‐month period, in which psychoeducation around sensitive parenting and discipline is shared as well as review of videotaped interactions between caregiver and child. The dummy intervention in this RCT consisted of phone calls and general discussion around their child’s development.

### Quality appraisal and Risk of Bias

The GRADE assessment process gave a *low‐quality* rating for all three outcomes, as quality was down rated due to the RoB and imprecision. There was an overall *high risk* or *some concern* about bias for all included studies according to the RoB2 rating tool. For most studies, neither participant or researchers could be blind to intervention arm. See supplementary materials for RoB (Supporting information S1: Table S4) and quality appraisal (Supporting information S1: Table S5) assessments.

### Outcomes and findings

*Caregiver sensitivity* was captured as an outcome measure in seven publications each relating to a different RCT and assessing effectiveness of the following interventions: ABC (Bick & Dozier, 2013; Raby et al., 2019), TCTP (Job et al., 2022), PCIT (Mersky et al., 2015), Child Directed Interaction Training (N’Zi et al., 2016), VIPP‐FC (Schoemaker, Juffer, et al., 2020), FFI (Van Andel et al., 2016). Caregiver sensitivity was a primary outcome measure for all studies except one (Raby et al., 2019), where caregiver sensitivity was being considered as a mediator for change in child receptive vocabulary development. They all used an observation (video or live) of interactions between the foster carer and foster child to rate the caregiver sensitivity to the child. Three studies use the dyadic parent–child interaction coding system (DPICS), in which every verbalisation from carer to child is coded and grouped into nurturing/positive and dysfunction/negative parenting behaviours. One study used the emotional availability scale, which assesses caregiver sensitivity as well as carer structuring, non‐intrusiveness, responsivity and involvement during interaction with the child (Van Andel et al., 2016). The remaining three studies reported coding sensitivity using likert scale measures of caregiver sensitivity as defined by Ainsworth (Bick & Dozier, 2013; Raby et al., 2019; Schoemaker, Juffer, et al., 2020).

The three interventions using DPICS were combined quantitatively in meta‐analysis. Two of these interventions considered PCIT, and one TCTP. The evidence favoured psychological intervention over SAU (*hedges g* = 3.68; 95% CI: 0.28–7.29; *p* = 0.03) for increasing the frequency of positive nurturing parenting behaviours. There was no statistically significant decrease in negative parenting behaviours when comparing psychological interventions with SAU. Within the subgroup analysis, the frequency increase in positive nurturing parenting was substantially greater in the two studies considering PCIT (*hedges g* = 6.35 and 4.23), than in the case of TCTP (*hedges g* = 0.55). Although not statistically significant, there is a decrease in negative parenting behaviours associated with the PCIT, and a small but statistically significant increase in negative parenting behaviours associated with TCTP. Mersky et al., 2015, which had the largest sample size of these studies did, however, report a large ES for PCIT, and statistically significant decrease in mean frequency of negative parenting (*hedges g* = −11.57; 95% CI:−13.02 to −10.12; *p* < 0.01).

For the remaining RCTs considering caregiver sensitivity, the ES was calculated for the measures reported. There was no evidence of an increase in caregiver sensitivity associated with VIPP‐FC (*hedges g* = 0.135, *p* = 0.6). There was a significant increase in caregiver sensitivity associated with ABC (*hedges g* = 0.68, *p* < 0.01), reported by Raby et al., 2019. Bick and Dozier also report a statistically significant (*p* = 0.05) increase in caregiver sensitivity associated with ABC, although it was not possible to calculate an ES from data available (Bick & Dozier, 2013). Finally, a significant increase in caregiver sensitivity (*hedges g* = 0.82, *p* < 0.05) was associated with FFI when compared with SAU (Van Andel et al., 2016).

*Caregiver stress* was reported in 7 articles, corresponding to 7 RCTs assessing effectiveness of IY(Conn et al., 2018), PCIT (Danko, 2014; Mersky et al., 2015; N’Zi et al., 2016), MTFC‐P (Fisher & Stoolmiller, 2008; Jonkman et al., 2017), FFI (Van Andel et al., 2016). Caregiver stress was a primary outcome measure in 5 included articles, and secondary outcome measure in 2 (Danko, 2014; Jonkman et al., 2017). Although these interventions target parenting behaviour/skills rather than stress reduction directly. All studies except Fisher & Stoolmiller, 2008 use the parenting stress index‐short form (PSI‐SF). The NOSI‐R, which is the Dutch translation of PSI‐SF was used in one case (Van Andel et al., 2016). Effect size was calculated for 5 articles and a meta‐analysis was conducted. It was not possible to calculate an ES for data available from Fisher & Stoolmiller, 2008 or van Andel et al., 2016.

Overall, there was no statistically significant effect of psychological interventions on caregiver stress. However, there was a small statistically significant reduction in caregiver stress identified in the subgroup analysis for PCIT; (*hedges g* = −0.5 [95% CI: −1.00 to 0.00], *p* = 0.05). One study comparing MTFC‐P to SAU reports a small but statistically significant increase in caregiver stress associated with the intervention (Jonkman et al., 2017). However, while not included in the meta‐analysis, Fisher & Stoolmiller, 2008 report a statistically significant decrease in caregiver stress associated with MTFC‐P. Caregiver stress in this paper was extracted from parent daily report interviews, as opposed to a standardised measure (Fisher & Stoolmiller, 2008).

**FIGURE 2:**
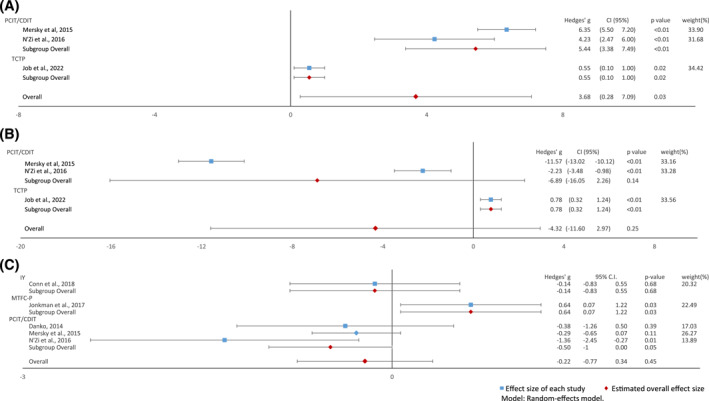
(A) Forest plot for positive parenting behaviours according to DPICS score. A higher score indicates greater frequency of behaviour. (B) Forest plot for negative parenting behaviours according to DPICS score. A lower score indicates lower frequency of behaviour. (C) Forest plot for parental stress according to PSI‐SF score. DPICS, dyadic parent–child interaction coding system; PSI‐SF, parenting stress index‐short form.

See Table 2 for summary data relating to caregiver sensitivity and caregiver stress, and Figure 2 for meta‐analysis forest plots.

**TABLE 2:** Tabulated findings for foster carer sensitivity and foster carer stress.

Study (author, year)	Intervention	Effect size *(Hedges g)*	95% confidence intervals	*p* value	*N (Int*: *Con)*
Negative or dysfunctional parenting as scored by DPICS					
Mersky et al., 2015 ^a^	PCIT	−11.57	−13.02 to −10.12	<0.01	83:46
N’Z et al., 2016 ^a^	CDIT	−2.23	−3.48 to −0.98	<0.01	7: 7
Job et al., 2022 ^a^	TCTP	0.78	0.32 to 1.24	<0.01	41: 36
Positive or nurturing parenting as scored by DPICS					
Mersky et al., 2015 ^a^	PCIT	6.35	5.50 to 7.20	<0.01	83:46
N’Zi et al., 2016 ^a^	CDIT	4.23	2.47 to 6.00	<0.01	7: 7
Job et al., 2022 ^a^	TCTP	0.55	0.10 to 1.00	<0.01	41: 36
Caregiver sensitivity					
Schoemaker, Juffer, et al., 2020 ^a^	VIPP‐FC	0.13	−0.37 to 0.65	0.6	30: 30
Van Andel et al., 2016 ^b^	FFI	0.82	Not reported	<0.05	65: 58
Raby et al., 2019 ^a^	ABC	0.67	0.25 to 1.11	0.02	45: 43
Bick & Dozier, 2013 ^b^	ABC	Hierarchical linear model indicates an increase in maternal sensitivity associated with the intervention		0.05	44: 52
Caregiver stress					
Conn et al., 2018 ^b^	IY	−0.14	−0.83 to 0.55	0.68	14: 17
Jonkman et al., 2017 ^a^	MTFC‐P	0.64	0.07 to 1.22	0.03	35:18
Danko, 2014 ^a^	PCIT	−0.38	−1.26 to 0.50	0.39	14: 7
Mersky et al., 2015 ^a^	PCIT	−0.29	−0.65 to 0.07	0.11	83: 46
N’Zi et al., 2016 ^a^	PCIT	−1.36	−2.45 to −0.27	0.02	7: 7
Van Andel et al. (2016)	FFI	Data not reported but paper reports no intervention effect on caregiver stress			65: 58
Fisher & Stoolmiller (2008)	MTFC‐P	Reports three level linear growth model indicating statistically significant reduction in caregiver stress in intervention group			57: 60

Abbreviations: ABC, attachment and biobehavioural catch‐up; CDIT, child directed interaction training; FFI, foster care—foster child intervention; IY(tf), trauma‐informed adapted incredible years; MTFC‐P, multidimensional treatment foster care for pre‐schoolers; PCIT, parent child interaction therapy; TCTP, triple P system for foster carers; VIPP‐FC, video‐feedback intervention to promote positive parenting and sensitive discipline in foster care.

^a^ Effect size, 95% CIs and *p* values calculated from M change and imputed SD change.

^b^ Findings as reported in paper.

*Placement stability* was reported by five articles (Blair, 2018; Fisher et al., 2005, 2009, 2011; Miller, 2007) relating to three separate RCTs. Blair, 2018, combines data from an RCT for PCIT (Mersky et al., 2015), with child welfare data. The primary outcome measure for the original RCT was caregiver stress and sensitivity. Fisher et al., 2005; Miller, 2007, both use data from the same RCT which is comparing EIFC with SAU. In this RCT the number of placement changes, and success or failure of permanent placements out of foster care (i.e., adoption or return to birth parents), were considered as placement outcome measures. Placement stability was the primary outcome reported in these articles, but the original RCT collected a range of other caregiver and child related outcomes. Fisher et al., 2009; Fisher et al., 2011 both utilise data from the same RCT and consider placement stability as a primary outcome measure. Fisher et al., 2009 consider success of permanent placement in the 24‐month period following intervention, while Fisher et al., 2011 considers placement disruption within foster care, defining this as placements where the child is moved at the request of the caregiver or because it is believed in the child's best interests, and not because of changes in foster carer circumstances, or a transition into permanent placement.

There is some evidence, based on two studies (Fisher et al., 2009, 2011), both utilising data from the same RCT, that MTFC‐P may reduce the risk of placement disruption or breakdown for children in foster care. Fisher et al., 2009, analyse placement outcomes for a subsample of children (*N* = 52) who had experienced four or more placements by the time of the trial. Twenty‐four months after baseline measurement a significantly greater proportion (*p* < 0.01) of the MTFC‐P group had achieved placement permenancy (defined as adoption or return to biological parent) than the regular foster care group, 69% and 30% respectively. There was also a significantly greater proportion (*p* < 0.01) of successful first permenant placement attempts for the MTFC‐P group. Fisher et al., 2011, explore placement disruption, within the full RCT sample (*N* = 117), and report a significant linear relationship between risk of placement disruption and challenging behaviour in the SAU but not intervention arm. The author's hypothesise that MTFC‐P may mitigate the impact of challenging behaviour on placement disruption. Miller, 2007 utilising a sub‐sample of participants data from within the EIFC trial (Fisher et al., 2005) considered various placement stability indicators including initiation of permanent placement and the frequency of placement disruption. They report statistically significantly (*p* = 0.017) fewer placement disruptions within 24 months for the start of the RCT in the IG. They also report that placement permenancy was more likely in the IG (Miller, 2007).

A similar approach integrating social care records with data from an RCT comparing PCIT with regular foster care (Mersky et al., 2015) demonstrated through logistic regression analysis that the odds of achieving placement permenance within 12 months was 2.63 times greater in the intervention compared with CG. This study did not find a significant difference in the proportion of placement disruptions between the two arms of the study (Blair et al., 2019).

## DISCUSSION

Previous systematic reviews and meta‐analysis have demonstrated the effectiveness of psychological interventions for children in foster care across childhood and adolescence (Bergström et al., 2020), and under 12 years of age (Hambrick et al., 2016). There is also evidence supporting the effectiveness of parenting interventions for foster carer skill with children and adolescence (Schoemaker, Wentholt, et al., 2020). However, this review focuses specifically on the caregiver and placement outcomes associated with psychological interventions targeting social, emotional and developmental needs of pre‐school children. To our knowledge it is the first review to focus solely on these outcome measures. This systematic review and meta‐analyses demonstrate effectiveness of psychological interventions for caregiver sensitivity and placement stability but not for caregiver stress. While these are promising findings the quality of evidence was low for all studies included and there was a high RoB identified for five studies. Study sample size was modest, increasing the uncertainty around findings. There is a high level of clinical and methodological heterogeneity across the studies. Finally, calculations used to estimate effect sizes rely on assumptions which introduce imprecision to the data. Caution should therefore be applied when interpreting these findings.

There are seven different therapeutic interventions described within this review. While these are distinct interventions in their materials, method of delivery, and length, they all focus on the relationship between the child and caregiver and are informed by similar theories, including attachment theory and social learning theory. The specific intervention targets vary, but generally aim to improve caregiver: child relationships, attachment, and child internalising or externalising difficulties. Psychoeducation and skills training for the foster carers were included within all interventions. In some cases (PCIT, TPTP, IY) this was delivered through group session, in others (ABC, FFI, VIPP‐FC) through individual ‘coaching’ sessions. MTFC‐P has the least in common with the other interventions included in the review as it is delivered over a year, whereas other interventions were generally delivered over a 2–4‐month period. It also includes both pre‐training for the foster carer and then individual support for child and foster carer. This therefore means that the time between pre‐ and post‐ intervention outcome measure for the MTFC‐P group is substantially longer than other interventions included in the review. While holding these differences in mind, this review considered all the interventions together with subgroup analysis and comparison between intervention types.

Providing care to a child in foster care requires specialised skills, including in many cases understanding of the potential impact of attachment difficulties or developmental trauma (Dozier, 2003; Vasileva & Petermann, 2018). Previous research has demonstrated the significance of caregiver sensitivity in relation to child behaviour (Cooke et al., 2022) and attachment (Deans, 2020). It can therefore be presumed that enhancing foster carer sensitivity will impact positively on the child and should be a core target for therapeutic intervention particularly for young children in foster care. This review finds that psychological interventions are effective in increasing caregiver sensitivity within this population. Comparing between interventions, the strongest evidence supports ABC and PCIT related interventions.

However, there are several factors limiting the reliability of comparison between interventions. Firstly, the conceptualisation of caregiver sensitivity is not consistent across different studies, with differing definitions and measurement approaches. Even within the three studies using the same outcome measure (DPICS) there were differences in the definition of ‘positive’ and ‘negative’ parenting behaviour when grouping the coded caregiver behaviour. Furthermore, there are many other aspects of caregiver skill and sensitivity (e.g. sensitive discipline) which were not considered in this review. Secondly, while the strongest evidence within the meta‐analysis was supporting PCIT, the measurement tool (DPICS) was developed for this intervention and may therefore be particularly sensitive to indicators of change associated with PCIT and less sensitive to other changes in caregiver sensitivity.

The second carer outcome variable reviewed is carergiver stress. This is important with regard to carers' health and wellbeing and also because carer stress levels can impact on their sensitivity toward the child, and commitment to continuing the child's placement (Goemans et al., 2020). Evidence regarding the effect of interventions on carergiver stress was inconsistent, and meta‐analysis concluded there was no effect of psychological interventions on caregiver stress. Findings were particularly inconsistent when comparing the effect of MTFC‐P on caregiver stress, with one study reporting a statistically significant increase in stress and another significant decrease.

While caregiver stress is reported as a primary outcome variable in five of the publications, the interventions themselves are not specifically targeting caregiver stress reduction. However, given the correlation between the child's difficulties and caregiver stress (Konijn et al., 2019), the limited benefit of these psychological interventions for caregiver stress was surprising. It could be that a reduction in caregiver stress is a longer‐term outcome and could not be captured by outcome measures taken immediately at the conclusion of the intervention. One hypothesis supporting this suggestion is that carers are being asked to learn and implement new skills and approaches during these interventions, and there could be an initial increase in stress and uncertainty for carers associated with implementing unfamiliar strategies. Another explanation consistent with the hypothesis that benefits for caregiver stress might be a longer‐term outcome is that if a reduction in caregiver stress is secondary to improvements in child behaviour and caregiver skill, then one would anticipate any reduction in stress coming after other changes had occurred. On the other hand, it might be that an increase in caregiver skill gained through interventions enables carers to ‘manage’ more challenging situations effectively, but that the level of stress to carers associated remains high or is increased as carers become more able. It is also likely that other factors influence foster carer stress levels specifically which may differn from other caregiver populations.

Finally, regarding placement stability there was some evidence that MCFC‐P and PCIT decrease the risk of placement disruption or increase the chance of placement permanency. However, there was a high level of variation in the way in which placement permanency was defined and measured across studies, limiting the extent to which data could be integrated within the review. This challenge has also been identified in other reviews considering placement stability (Maguire et al., 2024), and highlights a need to develop more consistent measurement of placement stability in order to assess intervention impact on this outcome. Furthermore, placement stability was only considered in a small number of published studies (two of the 5 included articles on placement stability were PhD theses rather publications in peer reviewed journals) and all bar one included study related to the MTFC‐P intervention and were completed through the same research team. This finding could also be relevant to understanding the impact of interventions on caregiver stress, as it might illustrate an increase in the threshold of complexity and distress that would result in placement breakdown following an intervention. Given the complex interaction between foster carer commitment, stress, placement stability and child outcomes, there is a need further research and longer term follow up measures (Turner et al., 2022, 2023).

Given the high level of heterogeneity across studies and multiple intervention types included it is difficult to draw firm conclusions regarding the comparative effectiveness of these interventions. This is particularly the case given that not all intervention types are considered for all outcome measures. Therefore, while PCIT and ABC show promise as interventions improving caregiver sensitivity, the potential benefit of MTFC‐P and IY for caregiver sensitivity is not assessed. Similarly, only 4 of the 7 intervention types are considered for caregiver stress and 2 of the 7 intervention types for placement stability. The low quality of included studies according to GRADE assessment and high RoB further limits the strength of evidence reported. Not all measures used have strong validity and reliability, particularly in relation to the various measures of caregiver sensitivity. There are also other limitations which need to be considered when interpreting findings from this review. Regarding inclusion criteria, RCTs available in English have been included in this review, and while grey literature was included where identified through database searches, specific grey literature databases were not included in the search strategy. A particular aim of this review was to focus specifically on early intervention (children age 7 or younger). However, a downside to this approach was the exclusion of other RCTs which included children 7 or under but also older children, for example, (Moody et al., 2020). As with any systematic review, limitations within the included articles also follow through into the review, and of relevance here was the common exclusion of certain groups including children with disabilities and the location of the included studies solely in high‐resource countries.

## CONCLUSION

There is evidence that psychological interventions improve caregiver sensitivity and may improve placement stability for children aged 0–7 in foster care. The impact of these interventions on caregiver stress is not conclusive. It is not possible to compare overall effectiveness across interventions given the variation in outcomes types, definitions and measures used across different studies. It remains important to consider the interaction between caregiver sensitivity, caregiver stress, placement stability and child outcomes, all previously demonstrated to influence one another (Konijn et al., 2019), in order to understand which interventions will be most effective and in which context. There is a need for larger and higher quality RCTs in this area, and for clarity and standardisation of the definition and measurement of these outcome variables, most notably in the case of placement disruption.

## AUTHOR CONTRIBUTIONS

**Camilla Biggs**: Conceptualization; formal analysis; methodology; project administration; writing—original draft; writing—review and editing. **Natalie Kirby**: Conceptualization; formal analysis; methodology; project administration; writing—review and editing. **Megan Garside**: Formal analysis; methodology; writing—original draft; writing—review and editing. **Gloria Cheung**: Formal analysis; methodology; writing—original draft. **Fiona Turner**: Conceptualization; supervision; writing—review and editing. **Nishant Jaiswal**: Methodology; supervision; writing—review and editing. **Helen Minnis**: Conceptualization; supervision; writing—review and editing.

## CONFLICT OF INTEREST STATEMENT

The authors declared no conflicts of interest.

## ETHICAL CONSIDERATIONS

Ethical approval is not applicable to this article as no new data were collected in this systematic review.

## TRIAL REGISTRATION

The systematic review was registered on the Prospective Register of Systematic Reviews (PROSPERO) database CRD 42022367449. Date of registration was the 25th November 2022. https://www.crd.york.ac.uk/PROSPERO/view/CRD42022367449.

## Supporting information

Supporting Information S1

## Data Availability

The data that supports the findings of this study are available in the supplementary material of this article.
