# Supplementary material for: Interventions for pre‐school children in foster care: A systematic review of the foster carer and system level outcomes from randomised controlled trials
Source: JCPP Adv. 2025 Nov 8;6(3):e70062. doi: 10.1002/jcv2.70062 (PMC13337127; doi:10.1002/jcv2.70062)
Supplement: Supplementary file 1 [file JCV2-6-e70062-s001.docx]

**Interventions for pre-school children in foster care: a systematic review of the foster carer and system level outcomes from randomised controlled trials**

**Supporting Information**

| ***Table S1: Search Strategy*** | |
| --- | --- |
| **Summary of Search Terms and development process** | 1. Randomised Controlled Trials, utilising the relevant search filter developed and published by Sottish Intercollegiate Guidelines Network (SIGN)(Scottish Intercollegiate Guidelines Network (SIGN). 2023). 2. Foster care setting, developed through author’s exploration of prior systematic reviews, and in particular including relevant items used within Turner et al, 2007 systematic review(Turner et al., 2007). 3. Children (0-7 years), utilising search items for Babies, Children and young people developed and published by the National Institute of Public Health for Quebec (INSPQ)(Tessier & Lacourse, 2023). |
| **Searches as completed for each database** | |
| **Embase 1947-Present, updated daily**  *Extracted 23/06/2023* | 1 Randomized Controlled Trial/ 792970  2 controlled clinical trial/ 469842  3 multicenter study/ 381029  4 Phase 3 clinical trial/ 70162  5 Phase 4 clinical trial/ 5491  6 exp RANDOMIZATION/ 100085  7 Single Blind Procedure/ 52285  8 Double Blind Procedure/ 213997  9 Crossover Procedure/ 75909  10 PLACEBO/ 415359  11 randomi?ed controlled trial$.tw. 329121  12 rct.tw. 54261  13 (random$ adj2 allocat$).tw. 55255  14 single blind$.tw. 32071  15 double blind$.tw. 251679  16 ((treble or triple) adj blind$).tw. 1944  17 placebo$.tw. 375098  18 Prospective Study/ 886681  19 Clinical Trial/ 1099294  20 or/1-19 2997179  21 Case Study/ 108721  22 case report.tw. 563927  23 abstract report/ or letter/ 1322362  24 Conference proceedings.pt. 0  25 conference abstract.pt. 4803490  26 Editorial.pt. 781034  27 Letter.pt. 1309953  28 Note.pt. 945649  29 or/21-28 8430819  30 20 not 29 2178212  31 babies.ti,ab,kf. 67852  32 baby.ti,ab,kf. 69620  33 infan*.ti,ab,kf. 687229  34 neonat*.ti,ab,kf. 440023  35 neo-nat*.ti,ab,kf. 634  36 newborn*.ti,ab,kf. 265108  37 new-born*.ti,ab,kf. 9388  38 perinat*.ti,ab,kf. 130911  39 hospitalized infant/ 1047  40 infant care/ 1755  41 high risk infant/ 3839  42 infant/ 812061  43 small for date infant/ 19707  44 prematurity/ 141518  45 premature.ti,ab,kf. 205955  46 large for gestational age/ 4830  47 newborn/ 695122  48 boy?.ti,ab,kf. 245032  49 boyfrien*.ti,ab,kf. 964  50 boyhood*.ti,ab,kf. 117  51 child*.ti,ab,kf. 2310733  52 child/ 2379140  53 child care/ 42305  54 preschool child/ 699691  55 school child/ 433266  56 fifth-grader*.ti,ab,kf. 556  57 first-grader*.ti,ab,kf. 671  58 fourth-grader*.ti,ab,kf. 472  59 girl/ 58970  60 girl?.ti,ab,kf. 252770  61 boy/ 46903  62 girlfrien*.ti,ab,kf. 693  63 girlhood*.ti,ab,kf. 44  64 juvenil*.ti,ab,kf. 126368  65 kid?.ti,ab,kf. 16923  66 kindergarten*.ti,ab,kf. 9812  67 minor?.ti,ab,kf. 354013  68 "minor (person)"/ 908  69 minority.ti,ab,kf. 110967  70 paediatric*.ti,ab,kf. 152085  71 pediatrics/ 102712  72 pediatric*.ti,ab,kf. 623331  73 pediatrics/ 102712  74 PICU.ti,ab,kf. 15719  75 preschool*.ti,ab,kf. 44464  76 preschool child/ 699691  77 pre-school*.ti,ab,kf. 8823  78 second-grader*.ti,ab,kf. 428  79 seventh-grader*.ti,ab,kf. 368  80 sixth-grader*.ti,ab,kf. 621  81 stepchild*.ti,ab,kf. 361  82 step-child*.ti,ab,kf. 81  83 stepchild/ 45  84 third-grader*.ti,ab,kf. 406  85 young*.ti,ab,kf. 1163953  86 toddler?.ti,ab,kf. 18699  87 (young adj2 person).ti,ab,kf. 2557  88 youngster.tw. 369  89 youth*.tw. 123320  90 juvenile/ 64857  91 nursery.ti,ab,kf. 12970  92 primary school.ti,ab,kf. 15508  93 reception.ti,ab,kf. 13500  94 primary school/ 17546  95 nursery/ 5649  96 nursery school/ 1774  97 or/31-96 6089373  98 exp Foster Home Care/ 5689  99 Foster care/ 5689  100 foster-care$.tw. 3218  101 (foster adj3 care$).tw. 4181  102 (foster adj3 parent$).tw. 975  103 (foster adj3 mother$).tw. 794  104 (foster adj3 father$).tw. 49  105 (foster adj3 child$).tw. 1839  106 ((kin or kinship or kindred) adj3 care$).tw. 561  107 ((kin or kinship or kindred) adj3 parent$).tw. 253  108 ((kin or kinship or kindred) adj3 mother$).tw. 79  109 ((kin or kinship or kindred) adj3 father$).tw. 31  110 ((kinship or kindred) adj3 child$).tw. 153  111 "living in care".tw. 402  112 "legal guardian".tw. 531  113 guardian*.tw. 14914  114 ("local authority" adj3 care$).tw. 150  115 (child adj3 placement).tw. 367  116 ("out of home" adj3 placement).tw. 361  117 ("out of home" adj3 care$).tw. 839  118 (alternat* adj3 care$).tw. 6289  119 ((relation or relative) adj3 care$).tw. 6537  120 or/98-119 38198  121 30 and 97 and 120 1896 |
| **Ovid MEDLINE(R) ALL <1946 to June 22, 2023>**  *Extracted 23/06/2023* | 1 Randomized Controlled Trials as Topic/ 162627  2 randomized controlled trial/ 595054  3 Random Allocation/ 106940  4 Double Blind Method/ 175500  5 Single Blind Method/ 32771  6 clinical trial/ 538195  7 clinical trial,phase i.pt. 24947  8 clinical trail, phase ii.pt. 0  9 clinical trial, phase iii.pt. 21784  10 clinical trial, phase iv.pt. 2419  11 controlled clinical trial.pt. 95340  12 randomized controlled trial.pt. 595054  13 multicenter study.pt. 335045  14 clinical trial.pt. 538195  15 exp Clinical Trials as topic/ 382851  16 or/1-15 1558509  17 (clinical adj trial$).tw. 477378  18 ((singl$ or double$ or trb$ or tripl$) adj (blind$3 or mask$3)).tw. 197474  19 PLACEBOS/ 35930  20 placebo$.tw. 246931  21 randomly allocated.tw. 36309  22 (allocated adj2 random$).tw. 40071  23 or/17-22 781328  24 16 or 23 1905416  25 case report.tw. 396191  26 letter/ 1220598  27 historical article/ 369327  28 or/25-27 1967134  29 24 not 28 1863014  30 exp Foster Home Care/ 3890  31 Foster care/ 3890  32 Child, Foster/ 191  33 foster-care$.tw. 2765  34 (foster adj3 care$).tw. 3514  35 (foster adj3 parent$).tw. 825  36 (foster adj3 mother$).tw. 616  37 (foster adj3 father$).tw. 51  38 (foster adj3 child$).tw. 1584  39 ((kin or kinship or kindred) adj3 care$).tw. 508  40 ((kin or kinship or kindred) adj3 parent$).tw. 219  41 ((kinship or kindred) adj3 mother$).tw. 37  42 ((kin or kinship or kindred) adj3 father$).tw. 28  43 ((kin or kinship or kindred) adj3 child$).tw. 231  44 "living in care".tw. 280  45 guardian*.tw. 10160  46 ("local authority" adj3 care$).tw. 113  47 (child adj3 placement).tw. 303  48 ("out of home" adj3 placement).tw. 332  49 ("out of home" adj3 care$).tw. 758  50 (alternat* adj3 care$).tw. 4688  51 ((relation or relative) adj3 care$).tw. 4978  52 or/30-51 28245  53 babies.ti,ab,kf. 41963  54 baby.ti,ab,kf. 44677  55 infan*.ti,ab,kf. 551213  56 neonat*.ti,ab,kf. 313486  57 neo-nat*.ti,ab,kf. 313  58 newborn*.ti,ab,kf. 197449  59 new-born*.ti,ab,kf. 5023  60 perinat*.ti,ab,kf. 91320  61 premature.ti,ab,kf. 141374  62 Infant, Extremely Premature/ 3866  63 Infant, Large for Gestational Age/ 4  64 Infant, Newborn/ 668485  65 Infant, Postmature/ 396  66 Infant, Premature/ 61379  67 Infant, Small for Gestational Age/ 8597  68 Infant/ 865370  69 boy?.ti,ab,kf. 166259  70 boyfrien*.ti,ab,kf. 747  71 boyhood*.ti,ab,kf. 92  72 child*.ti,ab,kf. 1672934  73 fifth-grader*.ti,ab,kf. 546  74 first-grader*.ti,ab,kf. 629  75 fourth-grader*.ti,ab,kf. 463  76 girl?.ti,ab,kf. 171616  77 girlfrien*.ti,ab,kf. 527  78 girlhood*.ti,ab,kf. 42  79 juvenil*.ti,ab,kf. 95893  80 kid?.ti,ab,kf. 10899  81 kindergarten*.ti,ab,kf. 8178  82 minor?.ti,ab,kf. 258733  83 minority.ti,ab,kf. 79514  84 paediatric*.ti,ab,kf. 85594  85 pediatric*.ti,ab,kf. 387461  86 PICU.ti,ab,kf. 6747  87 preschool*.ti,ab,kf. 35604  88 pre-school*.ti,ab,kf. 5880  89 second-grader*.ti,ab,kf. 402  90 seventh-grader*.ti,ab,kf. 356  91 sixth-grader*.ti,ab,kf. 593  92 stepchild*.ti,ab,kf. 291  93 step-child*.ti,ab,kf. 54  94 third-grader*.ti,ab,kf. 412  95 toddler?.ti,ab,kf. 13949  96 young*.ti,ab,kf. 819542  97 youngster.tw. 201  98 youth*.tw. 96308  99 Child/ 1910885  100 Child, Preschool/ 989207  101 (young adj2 person).ti,ab,kf. 1418  102 nursery.ti,ab,kf. 10660  103 primary school.ti,ab,kf. 11619  104 reception.ti,ab,kf. 10481  105 primary school/ 51241  106 nursery school/ 1506  107 girl/ 15181  108 boy/ 3953  109 or/53-108 4699229  110 29 and 52 and 109 1394 |
| **CINAHL**  *Extracted 23/06/2023* | **#** \| **Query** \| **Results** \|; S45 \| S10 AND S32 AND S44 \| 3,683 \|; S44 \| S33 or S34 or S35 or S36 or S37 or S38 or S39 or S40 or S41 or S42 or S43 \| 1,706,859 \|; S43 \| TX allocat* random* \| 16,090 \|; S42 \| (MH "Quantitative Studies") \| 35,635 \|; S41 \| (MH "Placebos") \| 13,711 \|; S40 \| TX placebo* \| 79,431 \|; S39 \| TX random* allocat* \| 16,090 \|; S38 \| (MH "Random Assignment") \| 79,552 \|; S37 \| TX randomi* control* trial* \| 261,901 \|; S36 \| TX ( (singl* n1 blind*) or (singl* n1 mask*) ) or TX ( (doubl* n1 blind*) or (doubl* n1 mask*) ) or TX ( (tripl* n1 blind*) or (tripl* n1 mask*) ) or TX ( (trebl* n1 blind*) or (trebl* n1 mask*) ) \| 1,262,393 \|; S35 \| TX clinic* n1 trial* \| 335,937 \|; S34 \| PT Clinical trial \| 113,337 \|; S33 \| MH "Clinical Trials+" \| 348,682 \|; S32 \| S11 OR S12 OR S13 OR S14 OR S15 OR S16 OR S17 OR S18 OR S19 OR S20 OR S21 OR S22 OR S23 OR S24 OR S25 OR S26 OR S27 OR S28 OR S29 OR S30 OR S31 \| 23,071 \|; S31 \| TI (relation or relative) N3 care$ OR AB (relation or relative) N3 care$ \| 5,415 \|; S30 \| TI alternat* N3 care$ OR AB alternat* N3 care$ \| 3,480 \|; S29 \| TI "out of home" N3 care OR AB "out of home" N3 care \| 890 \|; S28 \| TI "out of home" N3 placement OR AB "out of home" N3 placement \| 394 \|; S27 \| TI "child placement" OR AB local authority N3 care$ \| 287 \|; S26 \| TI local authority N3 care$ OR AB local authority N3 care$ \| 284 \|; S25 \| TI guardian* OR AB guardian* \| 4,225 \|; S24 \| TI "living in care" OR AB "living in care" \| 295 \|; S23 \| TI ( (kin or kinship or kindred) N3 child$ ) OR AB ( (kin or kinship or kindred) N3 child$ ) \| 286 \|; S22 \| TI ( (kin or kinship or kindred) N3 father$ ) OR AB ( (kin or kinship or kindred) N3 father$ ) \| 15 \|; S21 \| TI ( (kin or kinship or kindred) N3 mother$ ) OR AB ( (kin or kinship or kindred) N3 mother$ ) \| 27 \|; S20 \| TI ( (kin or kinship or kindred) N3 parent$ ) OR AB ( (kin or kinship or kindred) N3 parent$ ) \| 89 \|; S19 \| TI ( (kin or kinship or kindred) N3 care$ ) OR AB ( (kin or kinship or kindred) N3 care$ ) \| 562 \|; S18 \| TI foster N3 child$ OR AB foster N3 child$ \| 1,935 \|; S17 \| TI foster N3 father$ OR AB foster N3 father$ \| 39 \|; S16 \| TI foster N3 mother$ OR AB foster N3 mother$ \| 148 \|; S15 \| TI foster N3 parent$ OR AB foster N3 parent$ \| 888 \|; S14 \| TI foster N3 care$ OR AB foster N3 care$ \| 4,048 \|; S13 \| TI foster care OR AB foster care \| 4,450 \|; S12 \| TI foster-care* OR AB foster-care* \| 3,567 \|; S11 \| (MH "Foster Home Care") OR (MH "Foster Parents") OR (MH "Child, Foster") \| 6,777 \|; S10 \| S1 OR S2 OR S3 OR S4 OR S5 OR S6 OR S7 OR S8 OR S9 \| 1,280,771 \|; S9 \| (MH "Schools, Nursery") \| 1,268 \|; S8 \| MH(young person) \| 1,372 \|; S7 \| TI (young N2 (person)) OR AB ( young N2 (person)) \| 2,149 \|; S6 \| MH ("Child" OR "Child, Preschool") \| 585,018 \|; S5 \| AB (boy# OR boyfrien* OR boyhood* OR child* OR fifth-grader* OR first-grader* OR fourth-grader* OR girl# OR girlfriend* OR girlhood* OR juvenil * OR kid# OR kindergarten* OR minor# OR minority OR paediatric* OR peadiatric* OR pediatric* OR PICU OR preschool* OR pre-school* OR second-grader* OR seventh-grader* OR sixth-grader* OR stepchild* OR step-child* OR third-grader* OR toddler# OR young OR youngster* OR youth*) \| 706,321 \|; S4 \| TI (boy# OR boyfrien* OR boyhood* OR child* OR fifth-grader* OR first-grader* OR fourth-grader* OR girl# OR girlfriend* OR girlhood* OR juvenil* OR kid# OR kindergarten* OR minor# OR minority OR paediatric* OR peadiatric* OR pediatric* OR PICU OR preschool* OR pre-school* OR second-grader* OR seventh-grader* OR sixth-grader* OR stepchild* OR step-child* OR third-grader* OR toddler # OR young OR youngster* OR youth*) \| 550,796 \|; S3 \| MH ("Infant, Newborn" OR "Infant") \| 275,408 \|; S2 \| AB (babies OR baby OR infan* OR neonat* OR neo-nat* OR newborn* OR new -born* OR perinat*) \| 182,674 \|; S1 \| TI (babies OR baby OR infan* OR neonat* OR neo-nat* OR newborn* OR new-born* OR perinat*) \| 137,715 \| |
| **APA PsycInfo**  *Extracted 23/06/2023* | **Query** \| **Limiters/Expanders** \| **Results**; ReS91 \| S56 AND S78 AND S90 \| 449; S90 \| S79 or S80 or S81 or S82 or S83 or S84 or S85 or S86 or S87 or S88 or S89 \| 167,811; S89 \| TX allocat* random* \| 13,743; S88 \| (DE "Quantitative Methods") \| 3,963; S87 \| (DE "Placebo") \| 6,499; S86 \| TX placebo* \| 45,493; S85 \| TX random* allocat* \| 13,743; S84 \| DE "Random Sampling" \| 955; S83 \| TX randomi* control* trial* \| 59,247; S82 \| TX ( (singl* n1 blind*) or (singl* n1 mask*) ) or TX ( (doubl* n1 blind*) or (doubl* n1 mask*) ) or TX ( (tripl* n1 blind*) or (tripl* n1 mask*) ) or TX ( (trebl* n1 blind*) or (trebl* n1 mask*) ) \| 37,025; S81 \| TX clinic* n1 trial* \| 84,415; S80 \| PT Clinical trial \| 1,042; S79 \| DE "Clinical Trials+" OR DE "Randomized Controlled Trials" OR DE "Randomized Clinical Trials" \| 1,455; S78 \| S57 OR S58 OR S59 OR S60 OR S61 OR S62 OR S63 OR S64 OR S65 OR S66 OR S67 OR S68 OR S69 OR S70 OR S71 OR S72 OR S73 OR S74 OR S75 OR S76 OR S77 \| 25,272; S77 \| TI (relation or relative) N3 care$ OR AB (relation or relative) N3 care$ \| 3,720; S76 \| TI alternat* N3 care$ OR AB alternat* N3 care$ \| 1,929; S75 \| TI "out of home" N3 care OR AB "out of home" N3 care \| 1,447; S74 \| TI "out of home" N3 placement OR AB "out of home" N3 placement \| 1,104; S73 \| TI "child placement" OR AB local authority N3 care$ \| 279; S72 \| TI local authority N3 care$ OR AB local authority N3 care$ \| 221; S71 \| TI guardian* OR AB guardian \| 4,523; S70 \| TI "living in care" OR AB "living in care" \| 300; S69 \| TI ( (kin or kinship or kindred) N3 child$ ) OR AB ( (kin or kinship or kindred) N3 child$ ) \| 610; S68 \| TI ( (kin or kinship or kindred) N3 father$ ) OR AB ( (kin or kinship or kindred) N3 father$ ) \| 64; S67 \| TI ( (kin or kinship or kindred) N3 mother$ ) OR AB ( (kin or kinship or kindred) N3 mother$ ) \| 137; S66 \| TI ( (kin or kinship or kindred) N3 parent$ ) OR AB ( (kin or kinship or kindred) N3 parent$ ) \| 275; S65 \| TI ( (kin or kinship or kindred) N3 care$ ) OR AB ( (kin or kinship or kindred) N3 care$ ) \| 858; S64 \| TI foster N3 child$ OR AB foster N3 child$ \| 4,841; S63 \| TI foster N3 father$ OR AB foster N3 father$ \| 152; S62 \| TI foster N3 mother$ OR AB foster N3 mother$ \| 563; S61 \| TI foster N3 parent$ OR AB foster N3 parent$ \| 2,603; S60 \| TI foster N3 care$ OR AB foster N3 care$ \| 7,364; S59 \| TI foster care OR AB foster care \| 7,678; S58 \| TI foster-care* OR AB foster-care* \| 6,916; S57 \| DE "Foster Care" OR DE "Foster Parents" OR DE "Foster Children" OR DE "Foster Home Care" \| 8,816; S56 \| S46 OR S47 OR S48 OR S49 OR S50 OR S51 OR S52 OR S53 OR S54 OR S55 \| 1,227,305; S55 \| DE ("Child" OR "Child, Preschool") \| 194,816; S54 \| DE "Nursery School Students" OR DE "Nursery Schools" \| 971; S53 \| DE(young person) \| 20,742; S52 \| TI (young N2 person) OR AB ( young N2 person) \| 3,409; S51 \| DE ("Child" OR "Child, Preschool") \| 194,816; S50 \| AB (boy# OR boyfrien* OR boyhood* OR child* OR fifth-grader* OR first-grader* OR fourth-grader* OR girl# OR girlfriend* OR girlhood* OR juvenil * OR kid# OR kindergarten* OR minor# OR minority OR paediatric* OR peadiatric* OR pediatric* OR PICU OR preschool* OR pre-school* OR second-grader* OR seventh-grader* OR sixth-grader* OR stepchild* OR step-child* OR third-grader* OR toddler# OR young OR youngster* OR youth*) \| 1,053,707; S49 \| TI (boy# OR boyfrien* OR boyhood* OR child* OR fifth-grader* OR first-grader* OR fourth-grader* OR girl# OR girlfriend* OR girlhood* OR juvenil* OR kid# OR kindergarten* OR minor# OR minority OR paediatric* OR peadiatric* OR pediatric* OR PICU OR preschool* OR pre-school* OR second-grader* OR seventh-grader* OR sixth-grader* OR stepchild* OR step-child* OR third-grader* OR toddler # OR young OR youngster* OR youth*) \| 540,952; S48 \| DE ("Infant, Newborn" OR " Infant, Postmature" OR "Infant, Premature" OR "Infant") \| 47,792; S47 \| AB (babies OR baby OR infan* OR neonat* OR neo-nat* OR newborn* OR new -born* OR perinat*) \| 130,238; S46 \| TI (babies OR baby OR infan* OR neonat* OR neo-nat* OR newborn* OR new-born* OR perinat*) \| 59,166; S45 \| S10 AND S32 AND S44 \| 416; S44 \| S33 or S34 or S35 or S36 or S37 or S38 or S39 or S40 or S41 or S42 or S43 \| 163,208; S43 \| TX allocat* random* \| 13,743; S42 \| (MH "Quantitative Studies") \| 512; S41 \| (MH "Placebos") \| 2; S40 \| TX placebo* \| 45,493; S39 \| TX random* allocat* \| 13,743; S38 \| (MH "Random Assignment") \| 42; S37 \| TX randomi* control* trial* \| 59,247; S36 \| TX ( (singl* n1 blind*) or (singl* n1 mask*) ) or TX ( (doubl* n1 blind*) or (doubl* n1 mask*) ) or TX ( (tripl* n1 blind*) or (tripl* n1 mask*) ) or TX ( (trebl* n1 blind*) or (trebl* n1 mask*) ) \| 37,025; S35 \| TX clinic* n1 trial* \| 84,415; S34 \| PT Clinical trial \| 1,042; S33 \| MH "Clinical Trials+" \| 517; S32 \| S11 OR S12 OR S13 OR S14 OR S15 OR S16 OR S17 OR S18 OR S19 OR S20 OR S21 OR S22 OR S23 OR S24 OR S25 OR S26 OR S27 OR S28 OR S29 OR S30 OR S31 \| 24,200; S31 \| TI (relation or relative) N3 care$ OR AB (relation or relative) N3 care$ \| 3,720; S30 \| TI alternat* N3 care$ OR AB alternat* N3 care$ \| 1,929; S29 \| TI "out of home" N3 care OR AB "out of home" N3 care \| 1,447; S28 \| TI "out of home" N3 placement OR AB "out of home" N3 placement \| 1,104; S27 \| TI "child placement" OR AB local authority N3 care$ \| 279; S26 \| TI local authority N3 care$ OR AB local authority N3 care$ \| 221; S25 \| TI guardian* OR AB guardian* \| 5,282; S24 \| TI "living in care" OR AB "living in care" \| 300; S23 \| TI ( (kin or kinship or kindred) N3 child$ ) OR AB ( (kin or kinship or kindred) N3 child$ ) \| 610; S22 \| TI ( (kin or kinship or kindred) N3 father$ ) OR AB ( (kin or kinship or kindred) N3 father$ ) \| 64; S21 \| TI ( (kin or kinship or kindred) N3 mother$ ) OR AB ( (kin or kinship or kindred) N3 mother$ ) \| 137; S20 \| TI ( (kin or kinship or kindred) N3 parent$ ) OR AB ( (kin or kinship or kindred) N3 parent$ ) \| 275; S19 \| TI ( (kin or kinship or kindred) N3 care$ ) OR AB ( (kin or kinship or kindred) N3 care$ ) \| 858; S18 \| TI foster N3 child$ OR AB foster N3 child$ \| 4,841; S17 \| TI foster N3 father$ OR AB foster N3 father$ \| 152; S16 \| TI foster N3 mother$ OR AB foster N3 mother$ \| 563; S15 \| TI foster N3 parent$ OR AB foster N3 parent$ \| 2,603; S14 \| TI foster N3 care$ OR AB foster N3 care$ \| 7,364; S13 \| TI foster care OR AB foster care \| 7,678; S12 \| TI foster-care* OR AB foster-care* \| 6,916; S11 \| (MH "Foster Home Care") OR (MH "Foster Parents") OR (MH "Child, Foster") \| 959; S10 \| S1 OR S2 OR S3 OR S4 OR S5 OR S6 OR S7 OR S8 OR S9 \| 1,193,785; S9 \| (MH "Schools, Nursery") \| 1,247; S8 \| MH(young person) \| 255; S7 \| TI (young N2 (person)) OR AB ( young N2 (person)) \| 3,409; S6 \| MH ("Child" OR "Child, Preschool") \| 45; S5 \| AB (boy# OR boyfrien* OR boyhood* OR child* OR fifth-grader* OR first-grader* OR fourth-grader* OR girl# OR girlfriend* OR girlhood* OR juvenil * OR kid# OR kindergarten* OR minor# OR minority OR paediatric* OR peadiatric* OR pediatric* OR PICU OR preschool* OR pre-school* OR second-grader* OR seventh-grader* OR sixth-grader* OR stepchild* OR step-child* OR third-grader* OR toddler# OR young OR youngster* OR youth*) \| 1,053,707; S4 \| TI (boy# OR boyfrien* OR boyhood* OR child* OR fifth-grader* OR first-grader* OR fourth-grader* OR girl# OR girlfriend* OR girlhood* OR juvenil* OR kid# OR kindergarten* OR minor# OR minority OR paediatric* OR peadiatric* OR pediatric* OR PICU OR preschool* OR pre-school* OR second-grader* OR seventh-grader* OR sixth-grader* OR stepchild* OR step-child* OR third-grader* OR toddler # OR young OR youngster* OR youth*) \| 540,952; S3 \| MH ("Infant, Newborn" OR "Infant") \| 41; S2 \| AB (babies OR baby OR infan* OR neonat* OR neo-nat* OR newborn* OR new -born* OR perinat*) \| 130,238; S1 \| TI (babies OR baby OR infan* OR neonat* OR neo-nat* OR newborn* OR new-born* OR perinat*) \| 59,166 |
| **Cochrane Library**  *Extracted 23/06/2023* | #1 MeSH descriptor: [Foster Home Care] this term only 179  #2 MeSH descriptor: [Child, Foster] this term only 13  #3 (foster-care* OR foster care OR foster NEAR care OR foster NEAR parent OR foster NEAR mother OR foster NEAR father OR foster NEAR child):ti,ab,kw (Word variations have been searched) 1660  #4 (kin NEAR care OR kinship NEAR care OR kindred NEAR care OR kin NEAR parent OR kinship NEAR parent OR kindred NEAR parent OR kin NEAR mother OR kinship NEAR mother OR kindred NEAR mother OR kin NEAR father OR kinship NEAR father OR kindred NEAR father kin NEAR child OR kinship NEAR child OR kindred NEAR child):ti,ab,kw (Word variations have been searched) 89  #5 ("living in care" OR guardian OR "local authority" NEAR care OR child NEXT placement OR "out of home" NEAR placement OR "out of home" NEAR care OR alternative NEAR care):ti,ab,kw (Word variations have been searched) 7285  #6 ("living in care"):ti,ab,kw OR (guardian):ti,ab,kw OR ("local authority" NEAR care):ti,ab,kw OR (child NEXT placement):ti,ab,kw OR ("out of home" NEAR placement):ti,ab,kw (Word variations have been searched) 6058  #7 ("out of home" NEAR care):ti,ab,kw OR (alternative NEAR care):ti,ab,kw (Word variations have been searched) 1243  #8 #1 OR #2 OR #3 OR #4 OR #5 OR #6 OR #7 8909  #9 (boy*):ti,ab,kw OR (boyfrien*):ti,ab,kw OR (boyhood*):ti,ab,kw OR (child*):ti,ab,kw AND (fifth-grader*):ti,ab,kw (Word variations have been searched) 8180  #10 (first-grader*):ti,ab,kw OR (fourth-grader*):ti,ab,kw OR (girl*):ti,ab,kw OR (OR girlfriend*):ti,ab,kw OR (OR girlhood*):ti,ab,kw (Word variations have been searched) 8957  #11 (juvenil *):ti,ab,kw OR (kid):ti,ab,kw OR (kindergarten*):ti,ab,kw OR (minor):ti,ab,kw OR (minority):ti,ab,kw (Word variations have been searched) 27647  #12 (paediatric*):ti,ab,kw OR (peadiatric*):ti,ab,kw OR (pediatric*):ti,ab,kw OR (PICU):ti,ab,kw OR (preschool*):ti,ab,kw (Word variations have been searched) 78373  #13 (pre-school*):ti,ab,kw OR (second-grader*):ti,ab,kw OR (seventh-grader*):ti,ab,kw OR (sixth-grader*):ti,ab,kw OR (stepchild*):ti,ab,kw (Word variations have been searched) 1010  #14 (step-child*):ti,ab,kw OR (third-grader*):ti,ab,kw OR (toddler#):ti,ab,kw OR (OR young):ti,ab,kw OR (youngster*):ti,ab,kw (Word variations have been searched) 2395  #15 (youth):ti,ab,kw (Word variations have been searched) 9736  #16 (babies OR baby OR infan* OR neonat* OR neo-nat* OR newborn* OR new-born* OR perinat*):ti,ab,kw 93954  #17 (young NEAR person):ti,ab,kw 731  #18 MeSH descriptor: [Infant] this term only 28623  #19 MeSH descriptor: [Infant, Newborn] this term only 20279  #20 MeSH descriptor: [Child] this term only 69223  #21 MeSH descriptor: [Child, Preschool] this term only 35097  #22 MeSH descriptor: [Nurseries, Infant] explode all trees 13  #23 #9 OR #10 OR #11 OR #12 OR #13 OR #14 OR #15 OR #16 OR #17 OR #18 OR #19 OR #20 OR #21 OR #22 214116  #24 MeSH descriptor: [Clinical Trial] explode all trees 45348  #25 (Clinical Trial):ti,ab,kw 723656  #26 (Clinic* NEAR trial*):ti,ab,kw 543306  #27 (singl* NEAR blind*):ti,ab,kw OR (singl* NEAR mask):ti,ab,kw OR (doubl* NEAR blind*):ti,ab,kw OR (doubl* NEAR mask*):ti,ab,kw (Word variations have been searched) 396643  #28 (tripl* NEAR blind*):ti,ab,kw OR (tripl* NEAR mask*):ti,ab,kw OR (trebl* NEAR blind*):ti,ab,kw OR (trebl* NEAR mask*):ti,ab,kw (Word variations have been searched) 2918  #29 (randomis* control* trial*):ti,ab,kw (Word variations have been searched) 124429  #30 MeSH descriptor: [Random Allocation] this term only 23362  #31 (placebo*):ti,ab,kw (Word variations have been searched) 366717  #32 MeSH descriptor: [Placebos] this term only 25631  #33 (allocat* NEAR random*):ti,ab,kw (Word variations have been searched) 84684  #34 #24 OR #25 OR #26 OR #27 OR #28 OR #29 OR #30 OR #31 OR #32 OR #33 1088589  #35 #8 AND #23 AND #34 2056 |
| *Search rerun on 20.01.2025 identifying the following additional articles added to databases between 24.06.2023 and 20.01.2025: Embase: 323; Medline: 206; CINAHL: 348; PsycInfo: 49; Cochrane: 429* | |

***Table S2: Equations used to combine and impute data for analysis***

1. *Equations used to calculate change in mean and variance for meta-analysis.*

**Mean(change)**	$M(post)-M(pre)$

**SD( change)**	$\sqrt{{SDpre}^{2}+{SDpost}^{2}-(2x cor.coef. x SDpre x SDpost)}$

A correlation coefficient of 0.6 was calculated from data related to parental stress reported in one study (Conn et al., 2018). This was then used an estimated correlation coefficient for all studies, an approach suggested within the Cochrane handbook.

2. *Equations used to combine interventions groups in 3 arm studies.*

| ***Table S3: Demographic information for participants*** | | | | | |
| --- | --- | --- | --- | --- | --- |
| **Study (Author, year)** | **Child age** | **Child gender** | **Child ethnicity** | **Other child demographics** | **Caregiver demographics** |
| Bick & Dozier, 2013 | Mean age 9.9 months (SD=6.05, range 1-22 months) | 48% female | 59% African American, 28% White non-Hispanic, 6% Hispanic, 1% Asian American, and 6% biracial | N/A | Mean age 45 years (SD=10.7, range 24 to 74 years; 43% African American, 46% White non-Hispanic, 7% Hispanic, and 4% biracial |
| Blair, 2018 | Mean age 4.6 years (SD=1.31) | 56.9% female | Ethnicity, 56.1% African-American, 19.5% White, 12.2% Hispanic/Latino, 12.2% Other | N/A | Mean age 44.7 years (SD=11.12); 89.4% female; Ethnicity: 45.9% African-American, 48.4% White, 5.7% Other; Average length of time as a foster parent = 50 months (SD=69.02); 41.5% had a college degree, 36.4% attended some college, and 22% had a high school degree/GED. |
| Conn et al., 2018 | Intervention: Mean age 53.33 months (SD=16.81)  Control: Mean age 42.88 months (SD=12.59) | Intervention: 40% female  Control: 23.5% female | Intervention: 20% Black  Control: 52.9% Black | Intervention: Average age entering FC 33.14 months (SD=16.45); Average months in FC 19.07 (SD=15.72)  Control: Average age entering FC 17.94 months (SD=12.73); Average months in FC 24.29 (SD=18.25) | Intervention: 81.3% female; 18.8% Black; 25% aged 18-35, 62.5% aged 36-44, 12.5% aged over 45  Control: 93.8% female; 29.4% Black; 29.4% aged 18-35, 58.8% aged 36-44, 11.8% aged over 45 |

| Danko, 2014 | Mean age 3.56 years (SD=0.99, range 2.08-5.67) | 74.1% male | 66.7% African American, 11.1% Latino, 3.7% Caucasian, 3.7% Asian, 14.8% Multiracial | Mean child’s age at start of placement 1.93 years (SD=1.37, range 0.17-5) | 92.6% female; Mean age 44.11 years (SD=13.12 range 23-81); Ethnicity 66.7% African American, 14.8% Latino, 18.5% Caucasian; Average no years education 14.69 (range 4 - 22 years); Average household income between $30,001 and $40,000; 26.9% single, 3.8% cohabitating, 34.6% married, 3.8% separated, 15.4% divorced, and 15.4% widowed; Mean no. years as a foster parent 4.6 (SD=4.48, range 0.33-15). |
| --- | --- | --- | --- | --- | --- |
| Fisher & Stoolmiller, 2008 | Mean age at baseline 4.4 years (range = 4.3–4.5 years) | Intervention 49% male Control 58% male | 89% European American, 5% Latino, 5% Native American, 1% African American | N/A | Not reported |
| Fisher et al., 2005 | Intervention: Mean age at study start = 4.50 (0.86)  Control: Mean age at study start = 4.22 (0.74) | Intervention: 66% male  Control: 60% male | Intervention: Ethnicity: 79% white, 3% native american, 18% hispanic or latino  Control: Ethnicity: 92% white, 4% Native American, 4% Hispanic or Latino | N/A | Not reported |
| Fisher et al., 2011 | Intervention: Mean age = 4.54, SD= 0.86 (range 3.01-6.78)  Control: mean age= 4.34, SD= 0.83 (range 3.10-5.91) | Intervention: 49.1% male  Control: 58.3% male | Intervention: 82.5% Caucasian, 10.5% Hispanic, 5.3% native American, 1.8% African American  Control: 93.4% Caucasian, 3.3% Hispanic, 3.3% native America | Intervention: 19.3% entering foster care for the first time, 14% recentring foster care, 66.7% moved to new foster home  Control: 26.7% entering foster care for the first time, 18.3% recentering foster care, 55% moved to new foster home | Not reported |
| Fisher, 2009 | Age at first placement, mean = 2.42 years (SD = 1.32, range 0-5 years) | Male n=27 Female n=25 | Ethnicity = 90.4% European American | Mean number of transitions = 5.79 (SD = 1.66); Mean numbers of prior placement transitions: Intervention = 6.21 (SD=1.59) Control = 5.26 (SD=1.63) | Not reported |
| Job et al., 2020 | Intervention: Mean age 42.8 months (SD=18.1, range 24-91)  Control:  Mean age 50.6 months (SD=19.8, range 24-95) | Intervention:  43% female  Control:  54% female |  | Intervention: Mean duration of stay in current foster family 17.3 months (SD=8.3, range 3-25)  Control: Mean duration of stay in current foster family 18.2 months (SD=8.5, range 2-40) | Intervention: Mothers' mean age 40.4 years (SD=7.1, range 25-57); Fathers' mean age 44.8 years (SD=6.6, range 32-58)  Control: Mothers' mean age 43 years (SD=6.2, range 29-56); Fathers' mean age 45.4 years (SD=6.7, range 34-62) |
| Jonkman et al., 2017 | Mean age 63.51 months (SD= 12.11) | 64% male |  | Average age in months at first out of home placement 36.26 (SD=20.72) ; mean time in current family in months 6.54 (SD=13.57); |  |
| Mersky et al., 2016 | Mean age 4.6 years | 54% female | 70% racial and ethnic minorities (61% African American) | Mean number of children per household 2.96 | 89% female; 51% racial and ethnic minorities 52% married; Median length of experience as a foster parent 2 years |
| Miller, 2008 | Mean age in years 4.33 (range 3.00-5.92) | 44.9% female | 85.9% European American, 7.7% Hispanic or Latino, 5.1% Native American, 1.3% African American | Mean number of days in foster care at start of study 162.64 (range 20-860). Average number of transitions 3.68 (range 1-9 transition) |  |
| N'zi et al., 2016 | Mean age 5.2 years (range 2.0-7.5) | 50% female | Ethnicity 64% Caucasian, 22% African American, 7% Hispanic, 7% biracial | Mean length of placement 3.01 years (range 3 months - 7.5 years); 14% of children adopted, 29% in permanent guardianship, 43% temporary guardianship, 14% informal guardianship arrangements (outside of court or CWS involvement) | 86% grandmothers, 14% great-grandmothers, mean age 56.5 years (range 45.9-73.0); 7% less than high school education, 7% completed high school; 36% completed some college; 36% completed college, 14% graduate degree; Mean annual family income $40,304 (range $11,000-$80,000; median $35,000), 29% lived below the poverty line |
| Raby et al., 2019 | Intervention age at assessment 52.1 months (SD=9.1)  Control age at assessment 51.4 months (SD=8.7) | Intervention 46.7% female  Control 51.2% female | Intervention: 31.1% Caucasian, 46.7% African American, 8.9% Hispanic, 13.3% Other  Control: 25.6% Caucasian, 51.2% African American, 7.0% Hispanic, 16.3% Other |  | Intervention: Age at intervention 47 years; 86.7% female; 50% Caucasian, 43.2% African American, 4.5% Hispanic, 2.3% Other Control: Age at intervention 48 years; 97.7% female; 33.3% Caucasian, 50% African American, 7.1% Hispanic, 9.5% Other |
| Schoemaker et al., 2020 | Mean age 3.63 years (SD=1.35, range 1–6 years) | 45% male |  | Mean time living with current family = 27.56 month (SD=15.98, range 5-63 months) | Mean age 45.43 years (SD=7.42, range 31-61 years) 83% female, 73% non-kinship foster families |
| Van Andel et al., 2016 | Intervention: Age in months: M (SD): 19.8 (14.4)  Control: Age in months: M (SD): 17.9 (14.7) | Intervention: Male (49%)  Control: Male (51%) |  | Intervention: 77% in first or second placement; 85% in non-kinship foster care; 65% long-term placements; 93% maltreatment experience  Control: 88% in first or second placement; 83% in non-kinship foster care; 62% long-term placements; 89% maltreatment experience | Intervention: 68% no prior experience as foster carers, 58% other children within family, 79% contact with biological parents  Control: 63% no prior experience as foster carers, 74% other children within family, 88% contact with biological parents |

| ***Table S4: Risk of Bias as assessed using Cochrane Risk of Bias 2 (RoB2)*** | | | | | | |
| --- | --- | --- | --- | --- | --- | --- |
| ***Study (Author, Year)*** | ***Randomisation process*** | ***Deviations from intended interventions*** | ***Missing outcome data*** | ***Measurement of the outcome*** | ***Selection of reported results*** | ***Overall bias*** |
| *Bick & Dozier, 2013* | Some concerns | High Risk | Low risk | Low risk | Some concerns | High risk |
| *Blair, 2018* | Some concerns | High risk | Low risk | Low risk | Some concerns | High risk |
| *Conn et al., 2018* | High risk | High risk | High risk | Some concerns | Low risk | High risk |
| *Danko, 2014* | Low risk | Low risk | Low risk | Low risk | Some concerns | Some concerns |
| *Fisher et al., 2005* | High Risk | Low risk | Low risk | High risk | Some concerns | High risk |
| *Fisher & Stoolmiller, 2008* | Some concerns | Low risk | low risk | Some concerns | Low risk | Some concerns |
| *Fisher, 2009* | High risk | High risk | Low risk | Low risk | Some concerns | High risk |
| *Fisher et al., 2011* | Some concerns | Low risk | Some concerns | Low risk | Low risk | Some concerns |
| *Job et al., 2020* | Some concerns | Low risk | Low risk | High risk | Low risk | Some concerns |
| *Jonkman et al., 2017* | Some concerns | Low risk | Low risk | Some concerns | Low risk | Some concerns |
| *Miller, 2008* | Some concerns | Some concerns | Low risk | Low risk | Some concerns | Some concerns |
| *Mersky et al., 2016* | Low risk | Low risk | Some concerns | Low risk | Low risk | Some concerns |
| *N'zi et al., 2016* | Low risk | Some concerns | Low risk | Some concerns | Some concerns | Some concerns |
| *Raby et al., 2019* | Some concerns | Some concerns | Low risk | Low risk | Some concerns | Some concerns |
| *Van Andel et al., 2016* | Low risk | Some concerns | Low risk | Some concerns | Low risk | Some concerns |

***Table S5: GRADE Assessment of overall quality***

| **Outcome** | **Number of studies** | **Number of unique participants** | **Design** | **Risk of bias** | **Inconsistency of results** | **Indirectness of evidence** | **Imprecision** | **Publication Bias, all possible confounding, dose gradient response** | **Overall quality estimate** |
| --- | --- | --- | --- | --- | --- | --- | --- | --- | --- |
| ***Parental sensitivity*** | *7* | *587* | *RCT* | *-1* | *Heterogeneity explained* | *Direct evidence* | *-1* | *NA* | *Low Quality* |
| ***Parental stress*** | *7* | *488* | *RCT* | *-1* | *Heterogeneity explained* | *Direct evidence* | *-1* | *NA* | *Low Quality* |
| ***Placement stability*** | *5* | *330* | *RCT* | *-1* | *Heterogeneity explained* | *Direct evidence* | *-1* | *NA* | *Low Quality* |
